# Using Conformational Sampling to Model Spectral and Structural Changes of Molecules at Elevated Pressures

**DOI:** 10.1021/acs.jpca.4c08065

**Published:** 2025-02-12

**Authors:** Felix Zeller, Philipp Pracht, Tim Neudecker

**Affiliations:** † University of Bremen, Institute for Physical and Theoretical Chemistry, Leobener Str. 6, D-28359 Bremen, Germany; ‡ Interdisciplinary Center for Scientific Computing, Ruprecht-Karls Universität Heidelberg, Im Neuenheimer Feld 205, D-69120 Heidelberg, Germany; § Bremen Center for Computational Materials Science, University of Bremen, Am Fallturm 1, D-28359 Bremen, Germany; ∥ MAPEX Center for Materials and Processes, University of Bremen, Bibliothekstr. 1, D-28359 Bremen, Germany

## Abstract

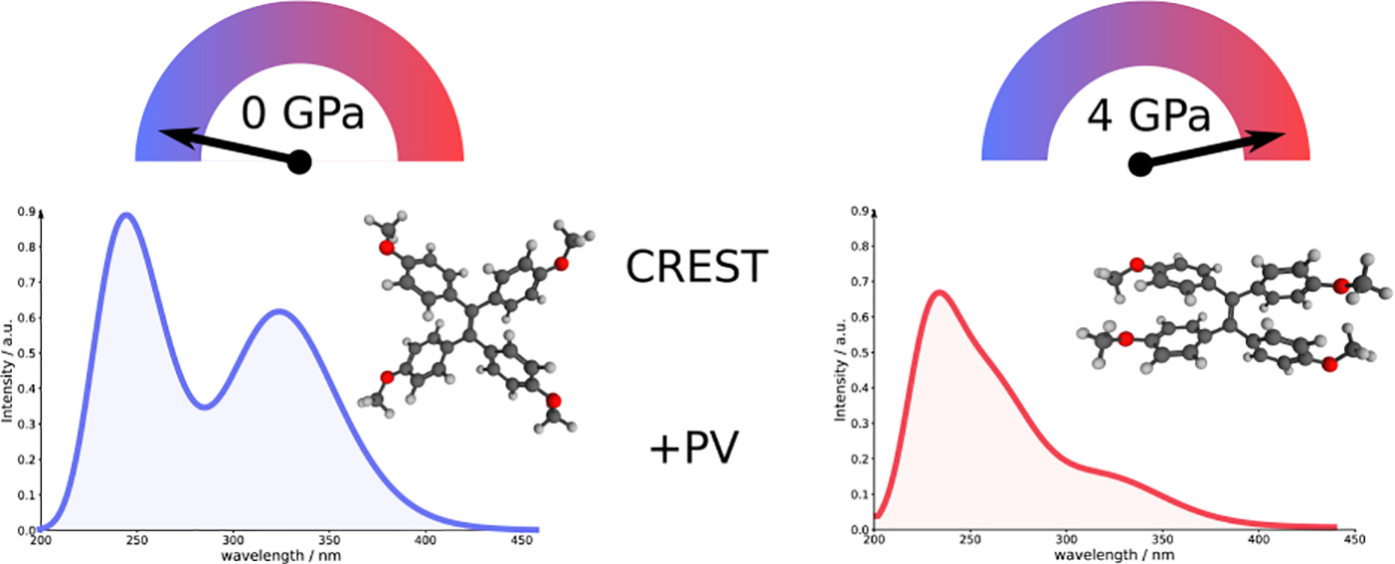

Conformational sampling is nowadays a standard routine in computational chemistry. Within this work, we present a method to perform conformational sampling for systems exposed to elevated pressures within the CREST program, allowing us to model pressure-induced changes of molecular ensembles and structural parameters. For this purpose, we extend the molecular Hamiltonian with the PV (pressure times volume) term, using the solvent-accessible volume. The volume computation is performed within the new standalone library libpvol. A first application shows good agreement with experimental data and provides a reasonable explanation for severe pressure-induced structural and spectroscopic changes of the molecules dichloroethane and tetra(4-methoxyphenyl)ethylene.

## Introduction

High-pressure chemistry, that is, chemistry occurring at pressures in the gigapascal (GPa) scale, offers a multitude of interesting phenomena. A nonexhaustive list contains effects such as phase transitions^1−3^ and crystal structure changes,^4−6^ amorphizations,^7,8^ changes in reaction behavior and kinetics,^9,10^ reversible changes in color,^11^ and the pressure-induced switching of the spin state of transition metal complexes.^12,13^ Experimentally, hydrostatic pressures up to several hundred gigapascals, as in planetary interiors,^14^ can be created using the diamond anvil cell.^15−17^

The experimental analysis of high-pressure phenomena can be combined with a plethora of theoretical models. Molecular dynamics (MD) simulations with a barostat can be used to simulate the behavior of amorphous materials, solutions, and biomolecules under high pressures.^18−28^ Pressure-induced crystal structure changes are routinely investigated using the plane-wave density functional theory (DFT) with the stress theorem.^29−38^ In both approaches, increasing pressure leads to shorter interatomic distances and thus higher electrostatic repulsion terms. Additionally, several models have been developed to approximate high-pressure effects in single-molecule calculations.^39−44^ Those usually lead to the potential energy surface (PES) being changed by an external pressure potential, which we therefore call the “pressure-modified” PES.

In recent years, the use of plane-wave DFT-based crystal structure search algorithms, the analogue of conformer sampling for periodic systems, led to an increasing accuracy in modeling high-pressure crystal structures.^4,45−47^ High-pressure MD simulations have been extensively used to model the pressure-induced denaturation process of proteins in the low GPa regime and show a high impact of pressure on the conformational ensemble.^26,28,48,49^

For single molecules at zero or ambient pressure, several sampling algorithms, e.g., based on MD^50,51^ or Monte Carlo techniques,^52,53^ have been developed to efficiently find the conformational ensemble on the PES.^54^ The iterative metadynamics-genetic crossing (iMTD-GC) algorithm forms the heart of the CREST^55−57^ program and offers an efficient and accurate way to find conformational ensembles of molecules and small molecular clusters, by using metadynamics together with Grimme’s extended tight-binding (xTB) methods.^58^ One of the many examples of the importance of conformational sampling includes trifluoro-pentanol dimers, where the correct description of the conformational ensemble was crucial to reproduce the experimental rotational spectrum.^59^

Within this publication, we aim to transfer the successes of high-pressure crystal structure search algorithms to single molecules. We hope this allows for a better understanding of high-pressure effects in solutions and amorphous materials and access to the fully developed toolkit of spectroscopic simulation techniques provided by modern quantum chemistry. For this purpose, we present a new library libpvol extending the CREST program, by adding the PV (pressure times volume) term to the electronic energy, allowing us to search the pressure-modified PES of single molecules for novel conformations and changes in the molecular conformational ensemble. We then use our approach to investigate the pressure dependence of the composition of molecular ensembles using the examples of the gauche/trans isomerization of dichloroethane (DCE) and the piezochromism of tetra(4-methoxyphenyl)ethylene (TMOE).

The rest of this paper is structured as follows. First, we discuss the details of the volume calculation and the implementation into the CREST program. Afterward, we will present the results of the chosen example applications.

## Methods

### Theory

To incorporate the effects of a pressure *P* into conformational sampling algorithms, the electronic energy *E*_*el*_ is extended with the PV term, and thus, the molecular enthalpy *H* is considered
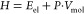
1where *P* is a constant user-defined pressure and *V*_mol_ is the solvent-accessible volume (SAV) of the molecule, which is obtained by the overlap of spheres around each atom having the solvent-accessible radius *r*_SA_ consisting of the van der Waals (vdW) radius *r*_vdW_ extended with a probe radius *r*_probe_
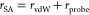
2

The evaluation of the SAV of proteins and other biomolecules is of longstanding interest, and consequently, numerous algorithms have been developed.^60^*V*_mol_ is routinely calculated by numerical grid integration of the solvent-accessible surface area (SASA), as is done for example in the XP-PCM.^44^
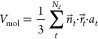
3

Here, *t* is a discretization point of the surface,  is its normal vector,  is its spatial position, *a*_*t*_ is its area element, and *N*_*d*_ is the number of discretization points. The discretization of the SASA is a standard procedure in continuum-based solvation models and can be achieved in several ways.^61−66^ In this work, a volume exclusion function *H*({}, ) is used on a Lebedev–Laikov grid to discretize the SASA,^67^ as introduced by Im et al.^63^ This procedure is also used in the recently presented analytical linearized Poisson–Boltzmann implicit solvation model.^68^*H*({}, ) implements a simple switching function that leads to a smooth SASA with an analytical gradient while being computationally efficient enough for seamless integration with semiempirical methods and other low-cost potentials. The volume exclusion function depends on the nuclear coordinates {} and takes the form of a product of atomic volume functions
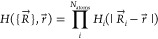
4

Using the substitution *x* = , *H*_*i*_(*x*) is given as
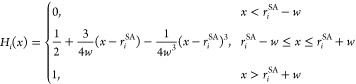
5Here, *r*_*i*_^SA^ is the solvent-accessible radius (eq 2) of atom *i* and *w* defines the switching region. *w* is chosen to be 0.3 Å, as recommended by Im et al.^63^ Each discretization point *t* belonging to an atom *j* is assigned an area *a*_*t*_ based on its position
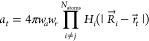
6where *w*_*a*_ and *w*_*r*_ are the angular and radial integration weights, respectively.

The volume gradient is obtained by derivation of eq 3 with respect to the atomic positions
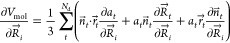
7

Note that the normal vectors  are independent of the atomic positions and the grid point coordinates  and only depend on one atomic position *k*, resulting in . This leads to the following simpler expression for the volume gradient
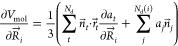
8where the second summation runs over all discretization points *N*_d_(*i*) associated with the atom *i*. Thus, the only additional quantity to compute is the derivative of the areas with regard to the atomic coordinates, which is implemented following the method of Im et al.^63^

In initial implementations, we attempted to use our extended hydrostatic compression force field (X-HCFF) model to conduct conformational sampling at elevated pressures.^57^ In the X-HCFF model, forces  are summed for each grid point *t* of the discretized vdW surface and added to the gradient of the associated atom *i*. The forces take the form^42^
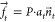
9

Unfortunately, the X-HCFF gradient does not fulfill the necessary condition of integrability, and it is not possible to define a clean energy term for it. However, inspecting eq 1, one can see that the X-HCFF gradient is equal to the second term in eq 8 times *P*. Thus, one can use the X-HCFF gradient as an approximation to the *PV*_mol_ gradient by assuming
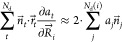
10

Using the X-HCFF gradient has the advantage that the derivatives of the area segments do not have to be computed, significantly reducing computation time. The validity of this approximation was tested by comparing the pressure-independent parts of X-HCFF and PV gradients for a test set containing the proteinogenic amino acids, where the X-HCFF gradients deviated by about 10–20% from the PV gradients. The coordinates, gradients, and gradient norms of the test set are provided in the SI. We generally advise using the analytical PV gradient; however, when dense integration grids are used, the computation of the PV term can become a computational bottleneck, where the usage of the X-HCFF gradient may save significant amounts of computation time.

### Implementation Details

The PV term described above is implemented in the standalone library libpvol, which is interfaced with the CREST program. This design allows for integration of the PV term into CREST via a modular calculator interface introduced in version 3.0.^57^ The libpvol library, similar in concept to the tblite package,^69^ is dedicated to the calculation of energy and gradient contributions. These contributions can be accessed through an interface object, enabling libpvol to be utilized in conjunction with any quantum mechanical software by extending the molecular Hamiltonian with an additional PV term.

The upcoming CREST version 3.1 includes integration of the PV functionalities by default. The libpvol library itself is written in Fortran, but Python and C++ wrappers are provided for ease of use, along with an interface to the atomic simulation environment (ASE).^70^ This flexible design ensures that libpvol can integrate with a variety of computational chemistry workflows.

Details on the use of wrappers and interfaces with libpvol can be found in its documentation.^71^ To allow refinement with quantum chemical accuracy and calculation of vibrational spectra at high pressure, we also implemented the PV computation into the Q-Chem software package. The implementation will be part of the Q-Chem 6.2.2 update.^72^ To enable fast frequency calculations, a seminumerical method was used, where the numerical Hessian of the PV gradient term is added to the analytical Hessian of the electronic energy.

**Figure 1:**
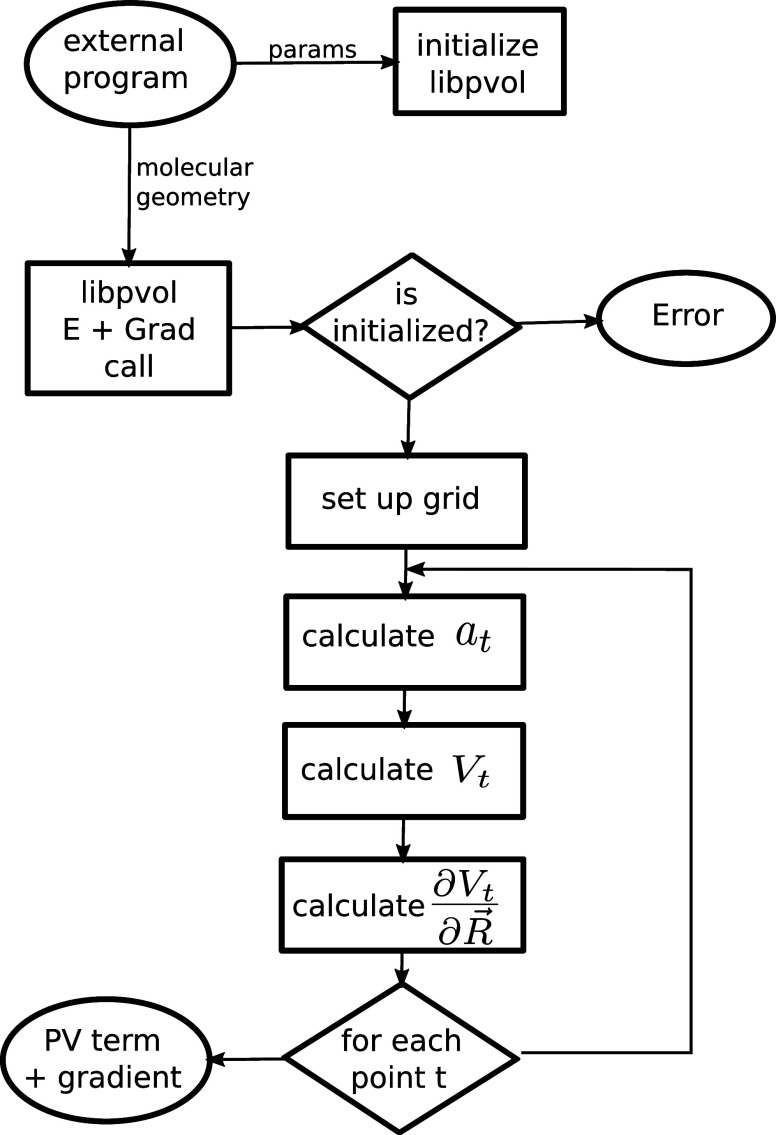
Schematic workflow for a pressure calculation with libpvol.

Figure 1 shows the structure of libpvol. In a first step, the interface has to be initialized with the calculation parameters. The most important parameters are the number of discretization points per atom, the set of vdW radii to use, and the probe radius *r*_probe_. Currently, the pairwise D3-cutoff radii for H–Pu and the Bondi set for H–Ar are implemented as possible sets of vdW radii.^73−75^ For the probe radius, a default value of 1.5 Å was chosen, which is approximately the radius of a water molecule. It should be noted that the added probe radius directly affects the SAV and consequently the energies added to conformers at a given pressure. We urge the user to employ conservative estimates of the probe radius.

After initialization, the PV term and its gradient can be computed. This requires only a molecular geometry as input. The evaluation of eqs 3–8 is implemented as a loop over discretization points *t*, which allows for efficient parallelization and memory usage. libpvol is available via GitHub.^71^

A PV calculation in CREST can be requested via the new TOML input format^76^ as shown in Listing 1 below.
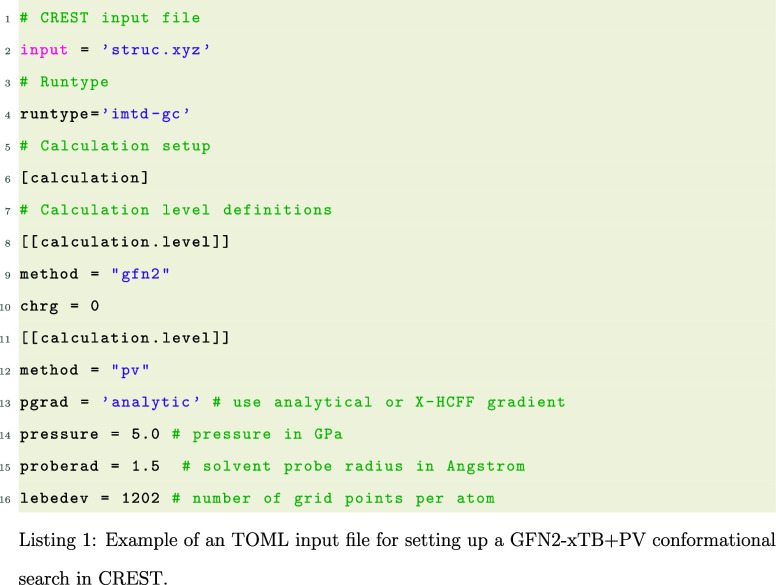


## Results

In the following, we present two applications of our workflow—the gauche/trans conformational ensemble of DCE and the spectroscopic darkening of TMOE at high pressure—and compare them with experimental data. The first application shows that one can model pressure effects on reaction equilibria and structural parameters reasonably well using a simple model such as the PV term. The second example demonstrates that new chemical insights can be gained via high-pressure conformational sampling.

### Gauche/Trans Isomerization of DCE

**Figure 2:**
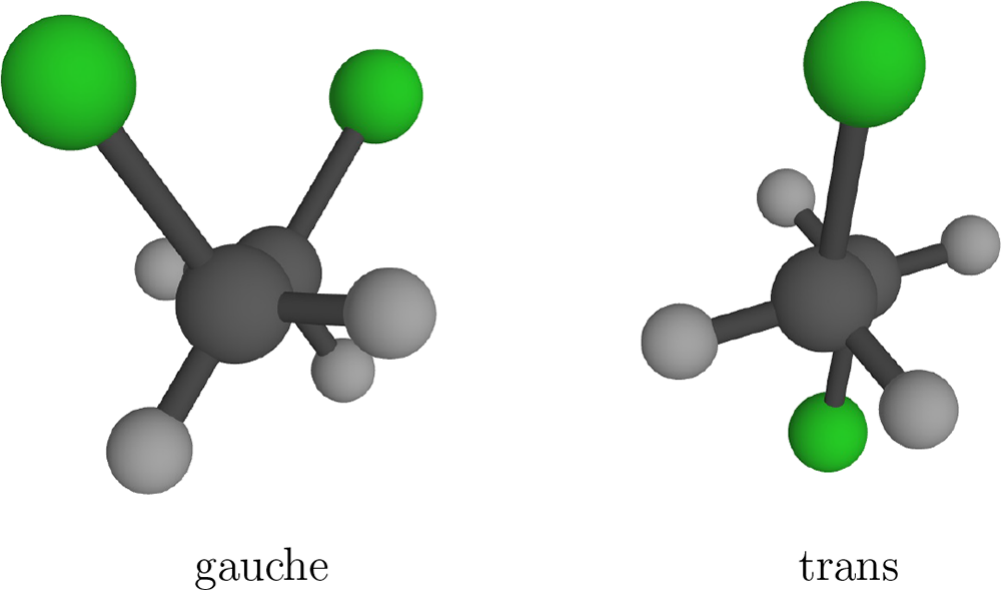
Conformations of DCE.

As a first example application, DCE was investigated, which, as commonly known, has two conformers: gauche and trans (Figure 2). The influence of pressure onto the equilibrium has been experimentally investigated by high-pressure infrared spectroscopy and ultrasound experiments.^77−80^ These studies coherently suggest the gauche conformer to become the predominant conformer of DCE at elevated pressures and presented reaction volumes Δ*V*_*g*/*t*_ between −0.7 and −5.9 cm^3^/mol, depending on the examined pressure range and the type of the experiment.

**Figure 3:**
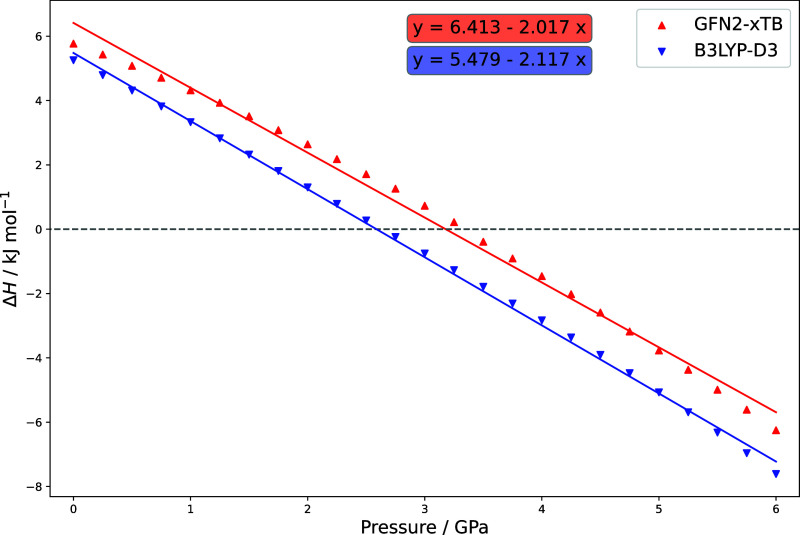
Enthalpy of isomerization from the trans to the gauche conformer of DCE between 0 and 6 GPa calculated at the B3LYP-D3BJ/def2-QZVP level of theory. The slope of the linear regression is interpreted as reaction volume Δ*V*_*g*/*t*_.

High-pressure conformational searches for DCE were performed using GFN2-*x*TB with the default probe radius of 1.5 Å at pressures between 0 and 6 GPa, otherwise using the default iMTD-GC workflow of CREST as described in detail in ref (55). Regardless of the input conformation, the conformational search correctly found solely the gauche and trans isomers at all investigated pressures. To obtain the molecular ensemble with a higher accuracy, the conformational search was refined with DFT geometry optimizations using the Q-Chem program package and the B3LYP-D3BJ/def2-QZVP^81−85^ level of theory with Becke–Johnson damping and the PV expanded Hamiltonian. The resulting gauche/trans isomerization enthalpies before and after refinement are shown in Figure 3. It can be seen that the DFT computations do not change the qualitative results but mostly will grant more accurate conformational energies. However, the effect here is rather marginal, as GFN2-*x*TB seems to describe the energy difference between the gauche and trans conformers rather well. In agreement with experiments, the gauche conformer becomes favored at elevated pressures. Assuming entropic effects to be negligible, Δ*V*_*g*/*t*_ can be approximated as the slope of the DFT plot to be −2.1 cm^3^/mol, which is in good agreement with the experimental data. It should be noted that the calculated reaction enthalpies do not depend on pressure in a strictly linear way, as eq 1 suggests. This behavior is caused by the dihedral angle between the two chlorine atoms slowly decreasing from 66.5° to 59.5°, allowing for an additional reduction in volume.

**Figure 4:**
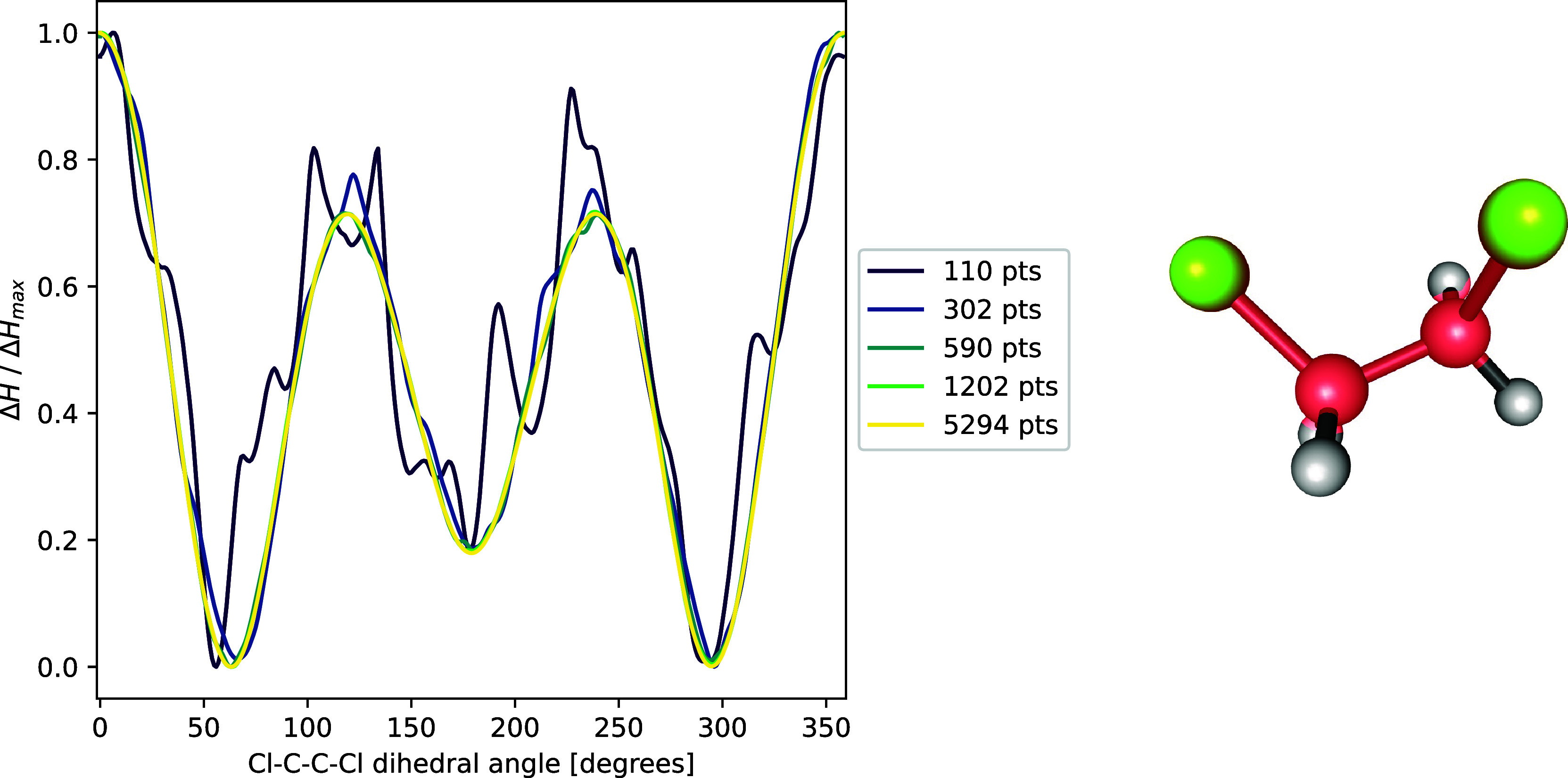
Normalized PES of DCE at 5 GPa cut along the Cl–C–C–Cl dihedral angle for differently dense integration grids.

It is important to note that when using small numbers of integration grid points, we found varying numbers of additional conformers. This behavior can be understood considering the PES cut along the Cl–C–C–Cl dihedral angle depicted in Figure 4. The low number of integration points leads to a large numerical error in the volume, causing the PES to become noisy and allowing for “artifact minima”. We generally found the numerical error to sufficiently decrease when more than 1000 points per atom are used. We thus set the default value to 1202 within libpvol and strongly advise against the use of fewer integration points.

### High-Pressure Spectra of TMOE

**Figure 5:**
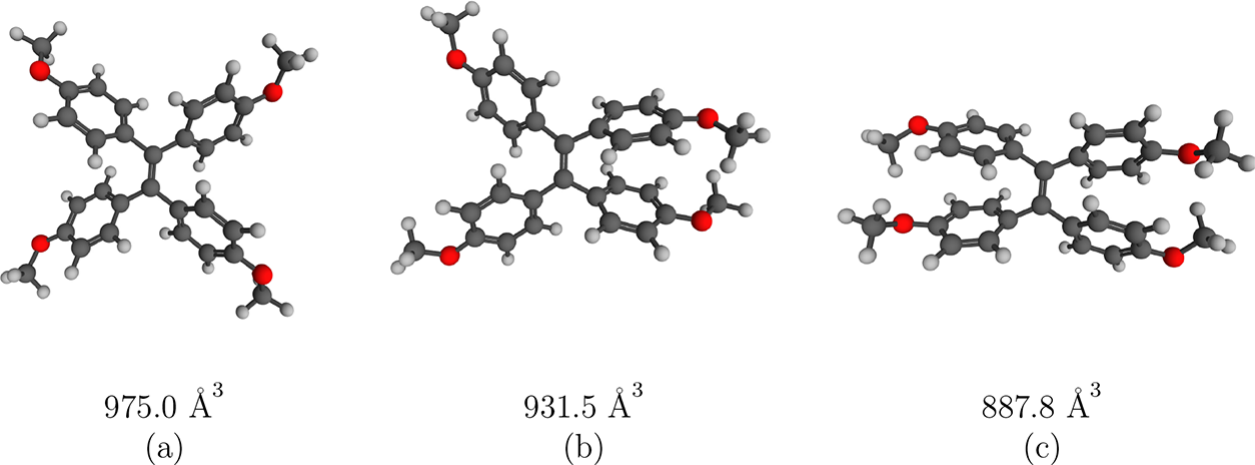
Predominant conformations of TMOE and their respective volumes in the absence of pressure (a), at 2.5 GPa (b), and at 4 GPa (c), obtained via conformation search. The respective Cartesian coordinates can be found in the SI.

TMOE is a propeller-shaped molecule consisting of four methoxyphenyl groups attached to a central ethene unit, as shown in Figure 5a. The spectroscopic behavior of TMOE was investigated by Wu et al. using high-pressure Raman and fluorescence spectroscopies up to 2.6 GPa.^86^ The molecule shows severe reductions in the spectral fluorescence and Raman intensities under elevated pressures. While the fluorescence intensities decline linearly, the Raman peaks are nearly unaffected up to 0.8 GPa and then start to quickly decline until they almost completely vanish at 1.7 GPa. Interestingly, the process was nearly completely reversible upon the relief of pressure. Based on constrained DFT optimizations and Raman simulations, Wu et al. suspected that the changes in the Raman spectrum are related to a change in the dihedral angle between the central ethylene unit and the phenyl groups, where an increase of the angle lowered the Raman intensities.

To investigate this behavior, conformation analysis was performed, using the default iMTD-GC workflow of CREST as described in detail in ref (55), at pressures between 0 and 4 GPa, using GFN2-*x*TB. To account for the experiments being carried out in the solid state, the probe radius was reduced to a more conservative value of 1.0 Å, resembling the closest distance to find a neighboring hydrogen atom. To get more reliable relative energies, the obtained ensembles were then refined with geometry optimizations at the B3LYP-D3BJ/cc-pVDZ^81−84,87−90^ level of theory and the PV term. In the absence of pressure, the ensemble of TMOE was found to consist of four propeller-shaped conformers (see Figure 5a), which only deviate in the orientations of the methoxy groups. Up to 2 GPa, the ensemble of TMOE is mostly unaffected by the pressure with similar Boltzmann weights and we found only small deviations in the orientation of the phenyl groups. However, at pressures above 2 GPa, the number of found conformers started to grow significantly, including a sandwich conformation (Figure 5c), which becomes the energetically favored conformation at pressures above 3.5 GPa. The number of found conformers spikes at 2.5 GPa with eight conformers having a Boltzmann weight larger than 1% and declines again at higher pressures. In the pressure range between 2 and 3.5 GPa, the ensemble is dominated by an intermediate conformer, where two phenyl units take the propeller and two take the sandwich form (Figure 5b). The isomerization to the sandwich conformer results in reduction in the molecular volume by about 10%, making it the only thermally accessible conformer at 4 GPa with a Boltzmann weight larger than 98%. The relative energies and Boltzmann weights of all conformers at the discussed pressures are provided in the SI. The absence of imaginary modes within frequency calculations confirmed that the new conformations are indeed minima on the pressure-modified PES. Also, we emphasize that a pressure-free geometry optimization of the sandwich conformer will restore the propeller shape, showing that external pressure can indeed be a contributing factor and lead to novel and unexpected conformations. However, contradicting the explanation of Wu et al., the dihedral angles between ethylene and the phenyl groups did not change significantly. As in the case of DCE, the refinement does not change the qualitative results of the original conformational sampling using GFN2-*x*TB. However, here, the aforementioned changes in the conformational ensemble appear at lower pressures from 1.5 GPa onward and the sandwich conformation becomes the most stable isomer at 2.75 GPa.

**Figure 6:**
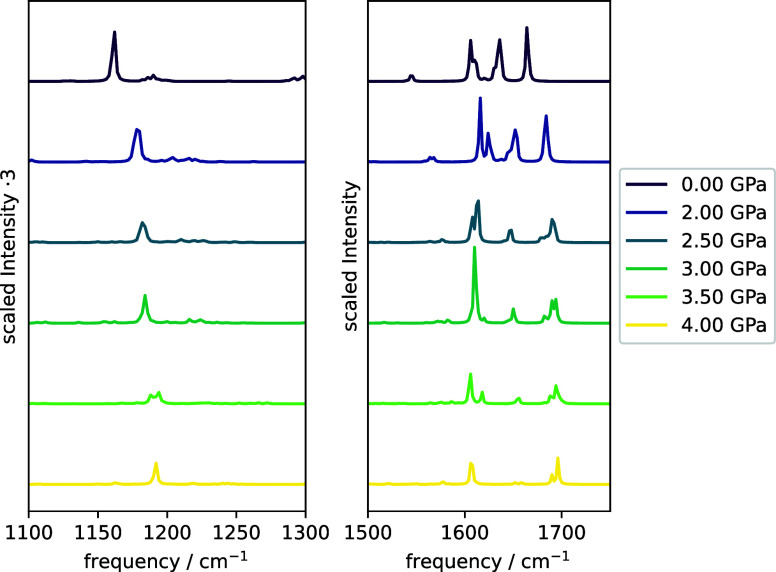
Boltzmann-weighted Raman spectra of TMOE ensembles in the region of the most intense peaks at pressures between 2 and 4 GPa. The intensities are scaled to the most intense peak of the pressure-free spectrum. For better visibility, the intensities in the left plot are scaled by a factor of 3. The spectra were calculated at the B3LYP-D3BJ/cc-pVDZ^87−91^ level of theory. The line spectrum was calculated from frequencies and oscillator strengths using Lorentzian functions with a broadening parameter of 1.0 cm^–1^. Information on the number of found conformers and respective Boltzmann weights can be found in the SI.

**Figure 7:**
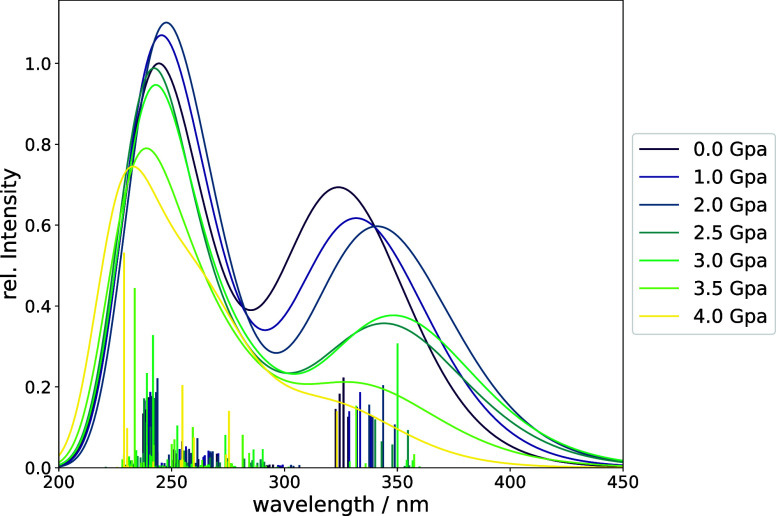
Boltzmann-weighted UV/vis spectra of TMOE ensembles at pressures between 0 and 4 GPa. The intensities are scaled to the most intense absorption at 0 GPa. The spectra were calculated at the CAM-B3LYP-D3BJ/aug-cc-pVTZ^84,87−91^ level of theory. The line spectrum was approximated using Gaussian functions with an exponential prefactor of 5.0 nm^2^. Information on the number of found conformers and respective Boltzmann weights can be found in the SI.

For further analysis, the Boltzmann-weighted Raman and UV/vis spectra for each TMOE ensemble were computed, which are shown in Figures 6 and 7, respectively. The Raman spectrum of TMOE exhibits two main features, an intense sharp peak at about 1130 cm^–1^ caused by the vibration of the carbon bond connecting the central ethylene unit and the phenyl rings and a broad split peak centered at approximately 1600 cm^–1^.^86^ These features are reproduced reasonably well by the calculated spectra, with a sharp peak at 1162 cm^–1^ and three neighboring peaks at 1606, 1636, and 1664 cm^–1^. For pressures up to 2 GPa, the peaks are found to be slightly red-shifted, but the peak intensities remain nearly unchanged. Upon isomerization to the intermediate and sandwich conformers, the peak intensities strongly decrease, and the splitting between the phenyl vibration modes increases. While our calculations do not suggest that the Raman spectrum becomes completely dark at elevated pressures as observed in the experiment, they show a strong decrease of the Raman intensities.

The experimental UV/vis spectrum of TMOE shows three absorption maxima at 230, 260, and 330 nm.^86^ The absorption spectra were simulated using time-dependent density functional theory (TD-DFT)^92^ using the CAM-B3LYP-D3BJ/aug-cc-pVTZ^87−91^ level of theory. Our excited-state calculations for the pressure-free ensemble show absorption maxima at 243 and 320 nm as shown in Figure 7. While the calculations do not reproduce the split UV maxima, they otherwise agree reasonably well with the experimental absorption spectrum.^86^ The presence of pressures up to 2 GPa already has an influence on the visible spectrum by causing a slight red shift and a decrease in the absorption intensity of the absorption maximum in the visible region. Analogous to the experiment, lower pressures already reduce the absorption intensities, while the Raman intensities remain nearly unaffected. At higher pressures, TMOE will isomerize to the sandwich conformation, which leads to the visible absorption spectrum of TMOE to darken nearly completely. Considering that an excitation wavelength of 365 nm was used in the experimental fluorescence spectra, the simulated absorption spectra explain the behavior in fluorescence experiments. The isomerization to the sandwich conformation leads to a strong reduction of absorption intensity around the used excitation wavelength and consequently will also strongly reduce the intensity in the fluorescence spectrum.

In conclusion, the high-pressure conformation analysis of TMOE provides a good qualitative explanation for its spectral darkening at high pressures. However, our results suggest that the pressures leading to this effect are overestimated by a factor of 2. We found the reversible spectral darkening to be caused by the isomerization from a propeller-shaped conformer to a sandwich-shaped conformer that is only stable at elevated pressures. Furthermore, the UV/vis spectra seem to be far more susceptible to small reorientations of the phenyl units, which explains a continuous darkening up to pressures of 1 GPa. Many conformations found at higher pressures are not stable in the absence of the same, clearly demonstrating the effects of directly including the pressure into the conformation sampling.

## Conclusions and Outlook

Within this article, we presented a method to include pressure effects in conformational sampling calculations. For this purpose, the molecular Hamiltonian is extended to the enthalpy by adding the PV (pressure times volume) term. Here, the pressure is kept constant and the volume is computed by integration of the SASA within a new standalone library libpvol, which is interfaced with the CREST conformational sampling program. We then used our implementation to model the gauche/trans isomerization of DCE and the spectral darkening of TMOE under elevated pressures. The results of the high-pressure conformational sampling agreed well with the experimental results for DCE. For TMOE, we provide a reasonable explanation for its spectral darkening, that is, it isomerizes to a new sandwich conformation that is only stable at elevated pressures.

We hope that our work will contribute to a better modeling of the influence of pressure on reactions in the GPa regime and help design novel molecules that change their spectroscopic properties when exposed to elevated pressures. In the future, we will work on the transfer of our approach to small molecular clusters and solvent shells. We also plan to postprocess our semiempirical results with more sophisticated single-molecule pressure models such as Gaussians on surface tesserae simulate hydrostatic pressure (GOSTSHYP).^39^

## Supporting Information Available

Test set to compare pressure-independent parts of X-HCFF and PV gradients (coordinates of test molecules are given in Å, gradients and norms in Bohr^2^); Cartesian coordinates of conformers in figure; relative energies in kJ/mol; and Boltzmann weights of conformers for weighted spectra of TMOE at each presented pressure (ZIP)
